# Impact of Catalytic Metals on the Ceramic Conversion Treatment of Ti-6Al-2Sn-4Zr-2Mo Alloy

**DOI:** 10.1021/acsomega.5c00209

**Published:** 2025-03-10

**Authors:** Zhenxue Zhang, Xiaoying Li, Hanshan Dong

**Affiliations:** School of Metallurgy and Materials, The University of Birmingham, Birmingham B15 2TT, U.K.

## Abstract

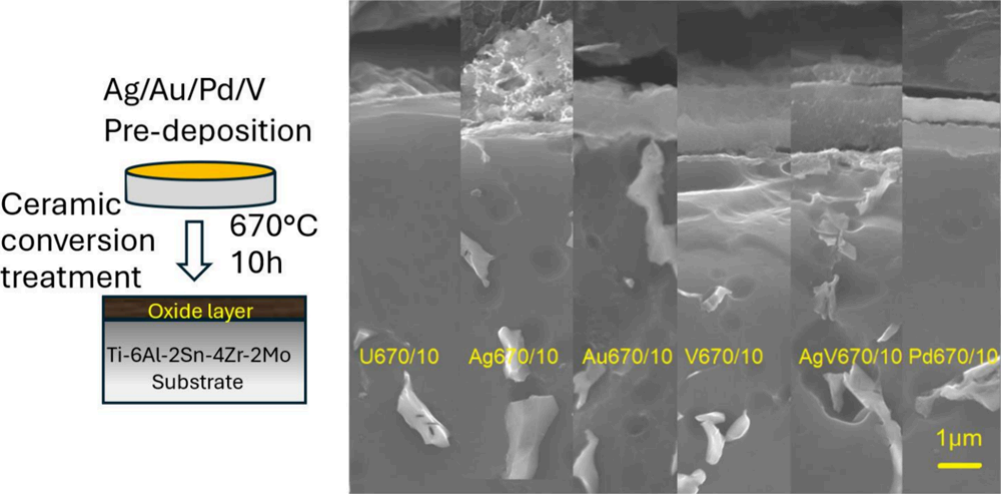

Ti-6Al-2Sn-4Zr-2Mo alloys (Ti6242) offer a high strength-to-weight ratio of up to 550 °C. Ceramic conversion treatment (CCT) is employed to enhance the titanium alloys with a ceramic layer and improve their poor tribological properties. However, longer treatment times and higher temperatures can have detrimental impacts on the materials. In this study, we predeposited various metallic layers like Ag, Au, V, Ag/V, Pd, and Ag/Pd on the surface of Ti6242 samples before CCT to compare their impact on catalyzing the treatment. CCT was carried out at 670 °C for 10 or 120 h. After treatment, the surface roughness/morphology, microstructure, and phase constituents were characterized. Surface hardness and nanohardness depth distribution were measured. Finally, reciprocating sliding tribological tests were carried out to study the friction and wear of the surface layers. CCT at 670 °C for 120 h created a compact oxide layer of less than 1 μm, which entitled the surface to high hardness and good tribological properties before the oxide layer was rubbed away. In a short treatment time of 10 h, vanadium was most effective in encouraging the oxidation of Ti6242, followed by Pd, Au, and Ag. Furthermore, Ag together with V or Pd boosted the growth of the oxide layer additionally. In the meantime, Ag, Pd, Au, and AgPd lowered the friction of the surface oxide layer and enhanced the wear resistance remarkably. In balance, gold helped to convert the surface of Ti6242 into a quality ceramic oxide layer efficiently with better tribological properties.

## 1 Introduction

Ti-6Al-2Sn-4Zr-2Mo (Ti6242) has a density of 4.54 g/cm^3^ and a tensile strength ranging from 897 to 1020 MPa, contributing to its high strength-to-weight ratio. The addition of the extra alloying elements in the titanium maintains its high resistance to creep, fatigue, and corrosion even at temperatures up to 550 °C.^1,2^ Ti6242 alloys find widespread use across industries in motorsport and aerospace, where they are employed in various applications such as engine parts and gas turbine components.^3^ However, the relatively low hardness and adhesive nature of titanium alloys often lead to seizure when they come into contact with counterpart materials under load.^4^ Unstable friction and low wear resistance necessitate further treatment to enhance performance in the service. Surface engineering techniques have been extensively utilized to maximize the performance of titanium alloys.^5,6^ However, there are relatively few reports on the modification of Ti6242 alloy, such as laser shot peening,^7^ nitriding,^8^ anodic oxidation,^9^ and the polymer derived ceramics (PDCs) method.^10^ Oxygen serves as a potent interstitial strengthening element and stabilizes the alpha phase in titanium alloys, making it suitable for surface strengthening. Anodic oxidation has been employed to improve the wear resistance of Ti6242; however, the resulting oxide film is often nonuniform and porous.^9^

Ceramic conversion treatment (CCT) at 600–700 °C in an oxygen-containing environment is an economical technique for enhancing the tribological performance of titanium alloys. This treatment forms a thin and compact oxide layer supported by a diffusion zone.^11^ However, prolonged treatment times at elevated temperatures may lead to recrystallization and adversely affect the mechanical properties. Recently, we discovered that silver and gold can accelerate the CCT of titanium alloys, making the process more efficient. Additionally, the incorporation of silver and gold into the oxide layer significantly reinforces the tribological behavior.^12,13^ Our earlier research indicates that silver is most effective on vanadium-containing Ti6Al4 V alloy,^12^ and a combination of Ag/Pd is even more proficient.^14^ Gold and vanadium demonstrate effectiveness on different titanium alloys such as commercial pure titanium (CPTi) and Ti6Al4 V alloy.^15,16^ Noble metals like Ag or Au have been blended into TiO_2_ composites for multifunctional applications, enhancing properties in various fields such as photocatalysis, photovoltaic, antibacterial, and mechanical applications.^17,18^ Ti6242 alloy exhibits greater oxidation resistance than CPTi and Ti-6Al-4 V.^19,20^ In our previous research, we found that gold played a beneficial role in enhancing the CCT of Ti6242.^21^ However, it remains unclear whether other metallic elements, such as Ag, Pd, or V, have any impact on the CCT of the Ti6242 alloy and its performance.

In this study, we predeposited a thin metallic layer (Ag, Au, V, Ag/V, Pd or Ag/Pd) on the surface of Ti6242 samples. CCT was carried out at 670 °C for 10 h to investigate if these metals can facilitate the ceramic conversion process compared to a normal CCT at 670 °C for 120 h, which we have successfully used to treat the Ti6242 racing car parts.^21^ The surface morphology and phase constituent changes were examined, and the oxide layer thickness was measured via cross-section SEM characterization. Mechanical properties, including hardness, friction, and wear, were investigated to assess the quality of the oxide layer.

## 2 Materials and Methods

**Figure 1:**
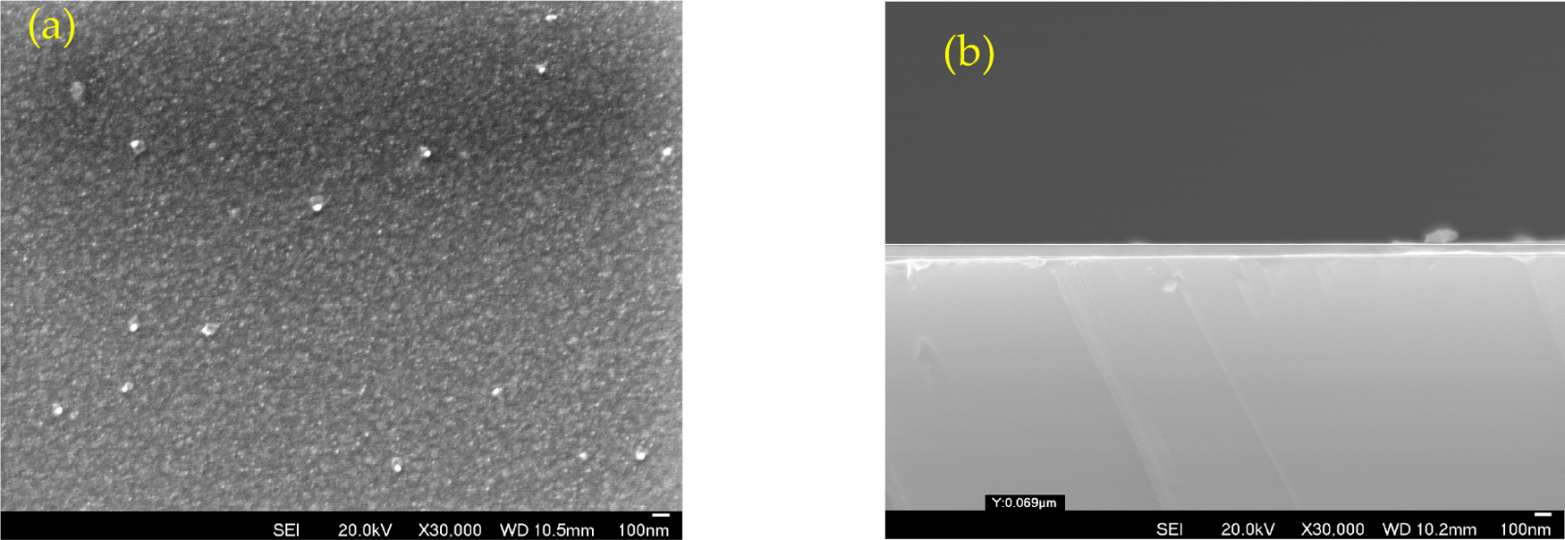
Ag/V layer deposited on silicon substrate by sputtering: (a) surface and (b) cross-section.

The material used in this research is Ti-6Al-2Sn-4Zr-2Mo supplied by Smiths High performance (UK) and its nominal chemical composition by weight is compared to a typical energy-dispersive X-ray analysis (EDX) measurement in Table 1. This alloy is a near alpha alloy with a small amount of beta phase. The rerolled round bar of Ti6242 was cut and sectioned into 4.5 mm thick coupons using SiC blades by a Metprep cutting machine. Coupons were progressively ground by sandpapers to 1200 grit and cleaned in an acetone bath. The gold layer (20–30 nm) was deposited on the cleaned Ti6242 coupons using a sputtering coater at 25 mA for 6 min. Ag (0.8*A*/3 min), V (1.5*A*/5 min), Ag/V (Ag 0.8*A*/V 1.5*A*/3 min), Pd (1*A*/4 min), and Ag/Pd (Ag 0.8*A*/Pd 1*A*/3 min) layers were deposited, respectively, via a Close Field Unbalanced Magnetron Sputtering Ion Plating equipment (Teer Coating Ltd., Droitwich, UK) to produce a thin metal layer of about 20–120 nm thickness (Table 2). A typical metallic layer of Ag/V can be seen in Figure 1.

**Table 1:** Chemical Composition (in wt %) of Ti-6Al-2Sn-4Zr-2Mo

element	Al	Sn	Zr	Mo	Si	Fe	O	C	N	Ti
nominal	5.5–6.5	1.75–2.25	3.5–4.5	1.75–2.25	0.1	Max 0.25	Max 0.12	Max 0.08	Max 0.05	bal.
EDX	6.03	1.85	3.85	1.48	0.23					86.55

**Table 2:** Sample Code and Detailed CCT Information

code	predeposition	temperature (°C)	duration (h)
Untreated			
U670–10		670	10
Ag670–10	Ag (50–80 nm)	670	10
Au670–10	Au (20–30 nm)	670	10
Pd670–10	Pd (40–80 nm)	670	10
V670–10	V (20–40 nm)	670	10
AgV670–10	AgV (50–100 nm)	670	10
AgPd670–10	AgPd (90–120 nm)	670	10
U670–120		670	120

Elite Thermal Systems Limited electric furnace was used to conduct the CCT. As prepared coupons were heated at a ramp rate of 8 °C/min to 670 °C for 10 or 120 h, and then they were cooled in the furnace. The sample code and their pretreatment and CCT details are listed in Table 2.

After CCT, the roughness of the surface was examined by an XP-200 Plus Stylus 3d-profilometer, and the surface morphology and elemental composition were studied (20 kV) by a JEOL 7000 scanning electron microscope equipped with an Oxford Aztec energy dispersive X-ray spectrometer (SEM/EDX, JEOL, Welwyn Garden City, UK). Phase constituents of the samples’ surface were identified by a PROTO AXRD benchtop power diffraction system (Proto, Crewe, UK), with a Cu source (*K*_α_ = 0.145 nm). The 2θ angles ranged from 20° to 80°, scanning at 40 kV voltage and 40 mA current. The diffraction peaks were identified by an X’Pert HighScore Plus program. The coupons were sectioned and mounted into conductive Bakelite for cross-sectional examination. A mirror-like finish was prepared in a three-step polishing process involving grinding and polishing under the lubricant of the activated colloidal silica. The cross-section of the sample was etched by Kroll’s reagent (2% HF + 10% HNO_3_ + balanced water) to reveal the microstructure and measure the oxide thickness under SEM.

A Mitutoyo MVK-H1 was employed to assess the surface microhardness via a Vickers indenter under a load of 50 g. An average of five to ten measurements was used as the value of microhardness. A Micromaterial nanoindenter was used to evaluate the nanohardness under the oxide layer.

A Phoenix TE-79 multiaxis tribology machine was employed to carry out the reciprocating friction and wear test. The counterpart WC ball had a diameter of 8 mm and slid at a speed of 5 mm/s under a load of 20 N for 1000 cycles over 5 mm. The test was repeated at least once to ensure accuracy. The XP-200 Plus TYLUS 3d-profilometer was used to plot the wear scars to inspect the width, depth, and thus area. Additionally, SEM and EDX were used to further scrutinize the track and the debris.

## 3 Results

### 3.1 Surface Appearance and Morphology

**Figure 2:**
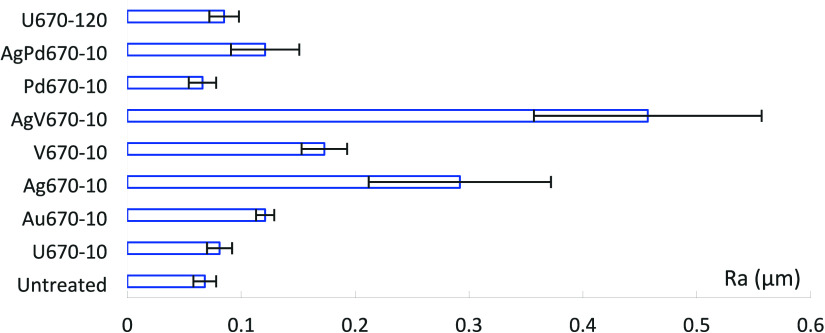
Surface roughness changes after CCT.

**Figure 3:**
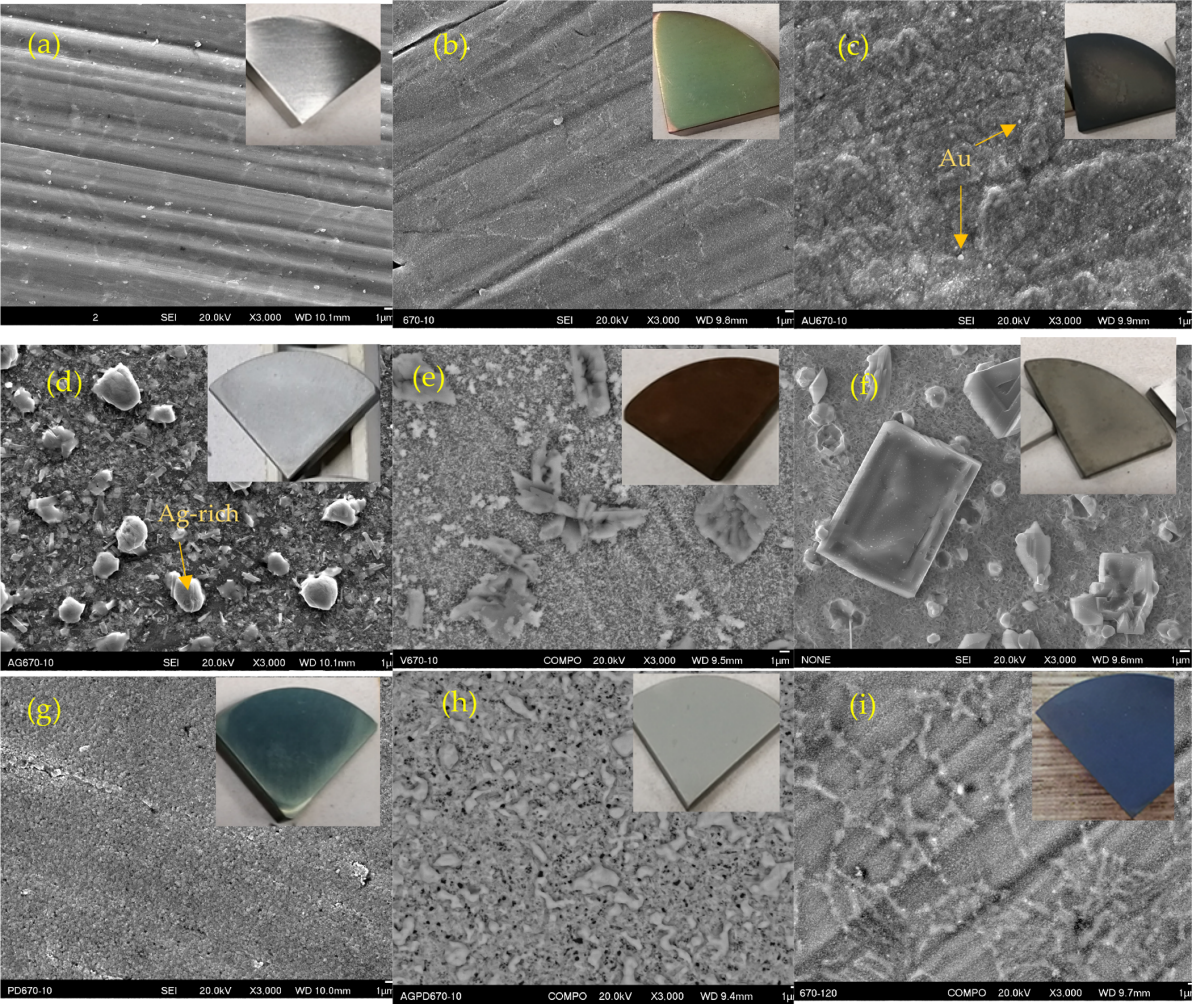
Surface morphology changes (SEM) of the Ti6242 samples (insets are photos of samples): (a) Untreated (b) U670–10, (c) Au670–10, (d) Ag670–10, (e) V670–10, (f) AgV670–10, (g) Pd670–10, (h) AgPd670–10, and (i) U670–120.

As shown in Figure 2, after 10 h CCT, the arithmetic average roughness (*R*_a_) value of the samples all increased. Sample Ti6242 turned from silver-gray metallic color (inset picture in Figure 3a) to yellow-green (Figure 3b). The Ra value of U670–10 increased slightly, and the grooves left after grinding were still discernible. For the gold-predeposited samples, the grinding mark became blurred after CCT, and small gold particles (identified in Figure S1) spread on the surface evenly which made the surface *R*_a_ value larger (Figure 3c). Large particles and agglomerates appeared on the surface of Ag670–10 which were silver-rich (Figure S2a), and the sample turned into silver-gray color with a much uneven surface (Figure 3d). Sample V670–10 took a brown color with some irregular shape blocks, which are rich in V and Al (Figure S3a), emerging on the surface (Figure 3e). The lumps turned into different-sized regular blocks with the assistance of Ag and V (sample AgV670–10 in Figure 3f), and the sample appeared dark gray with a very bumpy surface with the highest Ra value (Figure 2). Sample Pd670–10 looked very smooth with a dark blue color (Figure 3g), which is rich in Pd (Figure S5a). However, the sample AgPd670–10 (Figure 3h) showed a gray color with various sized particles on the surface, resulting in a higher Ra value compared to sample Pd670–10. Extending the treatment time to 120 h, the surface of U670–120 turned bluish (Figure 3i), and *R*_a_ value did not change too much in comparison with U670–10.

### 3.2 Cross-Sectional Microstructure

**Figure 4:**
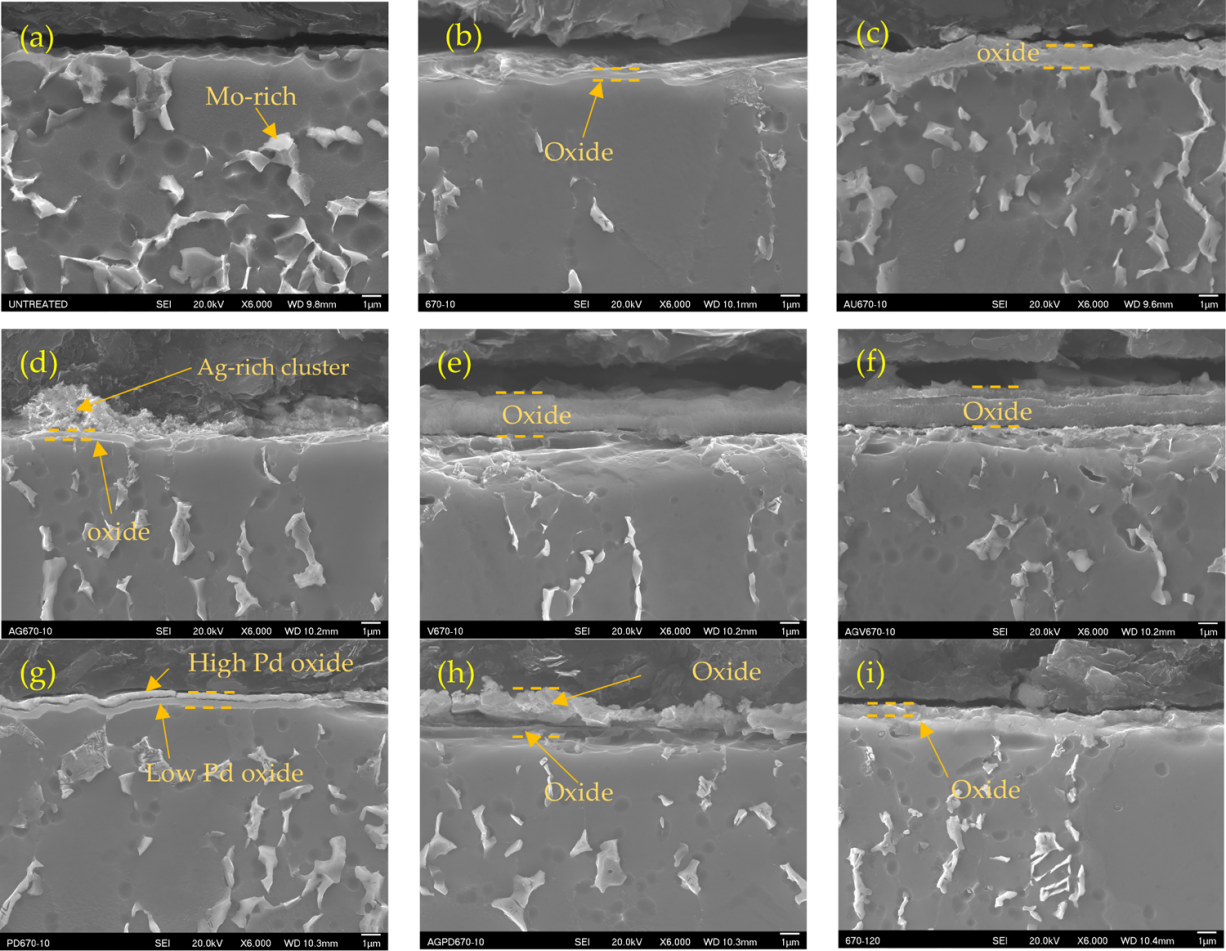
Cross-sectional SEM image of the Ti6242 samples: (a) Untreated, (b) U670–10, (c) Au670–10, (d) Ag670–10, (e) V670–10, (f) AgV670–10, (g) Pd670–10, (h) AgPd670–10, and (i) U670–120.

There are generally two zones in the untreated Ti6242 alloy, the gray area (alpha zone) and light areas containing more beta-stabilizing element of Mo (Figure 4a). After 10 h of CCT at 670 °C, a very thin oxide layer (0.2–0.3 μm) was formed on the surface of the Ti6242 sample (Figure 4b). The oxide layer grew to 0.6–0.9 μm when extending the treatment time to 120 h (Figure 4i). The O:Ti atomic ratio was slightly lower than 2 (Figure S7), suggesting an oxygen-deficient titanium dioxide was formed. With a gold predeposited layer, the oxide layer for Au670–10 became much thicker (0.7–0.9 μm) after the same CCT (Figure 4c). However, a very thin oxide layer with some large clusters, which were rich in silver, was produced on the surface of Ag670–10 (Figures 4d and S2b). A much thicker oxide layer of about 2.5 μm was formed on the surface of sample V670–10 (Figure 4e), while the oxygen content is relatively low in atomic composition (Figure S3). Ag/V further increased the oxide layer thickness and assisted in forming a multilayered structure (Figure 4f). The top layer was richer in oxygen than that of the underlayer (Figure S4b).

**Figure 5:**
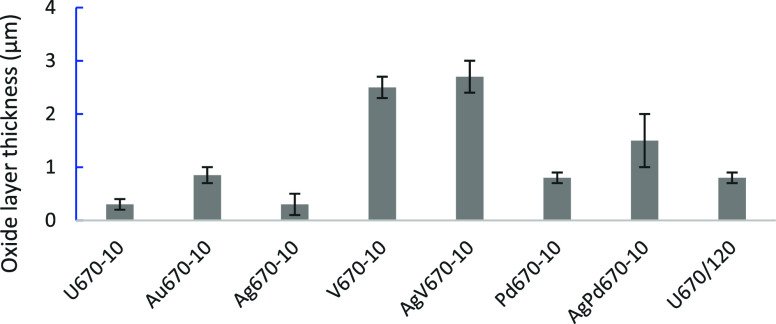
Thickness of the oxide layers on the CCTed Ti6242 samples.

Pd led to a dual layer with a Pd-rich top layer (36 at %) and a dense under layer with less Pd (7.6 at %) on the surface of Pd670–10 (Figures 4g and S5b). Mixed Ag and Pd also generated dual layers with a thick irregular superficial layer rich in Ag and Pd and a dense underlayer (Figures 4h and S6). The thickness of the oxide layer was measured at various locations via the scale in the SEM software, as compared in Figure 5. V and AgV speed up the oxidation most, followed by a mixture of Ag and Pd. Pd promotes thick double layers while gold helps to obtain a thick and dense oxide layer. Silver displays a less obvious effect on the ceramic conversion process. For most of the samples, the zones under the oxide layer (about 5 μm) have less/smaller beta phase in comparison to that of the untreated sample in Figure 4a, though they are very narrow. For Au670–10, there was not much change under the oxide layer in comparison to the untreated Ti6242 sample.

### 3.3 Surface Phase Constituents Evolution

**Figure 6:**
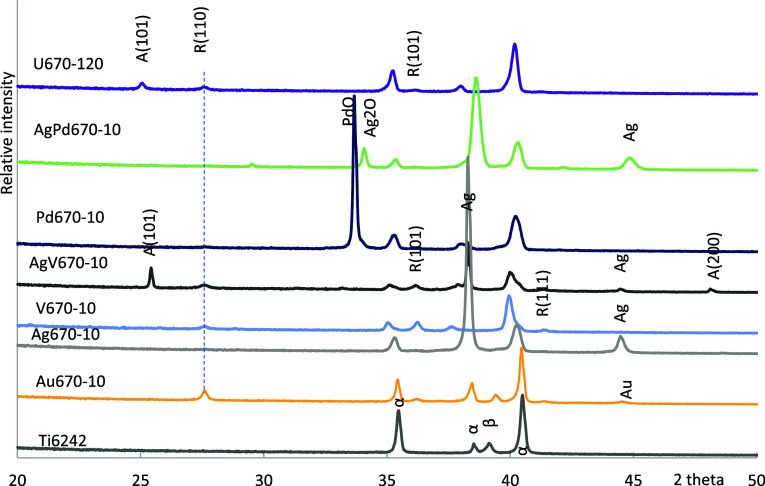
XRD identification of the surface phase constituents.

Ti6242 is a near-alpha titanium alloy composed of a small number of beta phases, as displayed in Figure 6. After CCT for 10 h at 670 °C, there was hardly any peak of oxide on the surface of U670–10 (Figure S8), the alpha peaks broadened and shifted toward lower angles. Both anatase and rutile phases were detected after 120 h of treatment (U670–120). Meanwhile, the alpha phases were still visible and shifted toward the lower angle.

With the assistance of gold, rutile TiO_2_ was identifiable, but anatase was hardly detectable. For sample Ag670–10, the oxide peaks were less clear, while the Ag peaks were significant, which might relate to the silver clusters in the surface region (Figure S2). There was rutile titanium dioxide appearing on the surface of V670–10, and some peaks were observed near to the left of alpha peaks. The higher V and Al contents detected by EDX in Figure S3 suggested some complex oxides were formed. A mixture of Ag and V helped anatase growth together with rutile on the surface of AgV670–10. Apart from rutile on the surface of Pd670–10, PdO was also generated. The titanium oxide peaks were less clear for sample AgPd670–10, but Ag_2_O was found on the surface. The strong alpha phase peaks indicate a shallow oxide layer formed on these samples.

### 3.4 Surface Hardness and Cross-Sectional Hardness Profile

**Figure 7:**
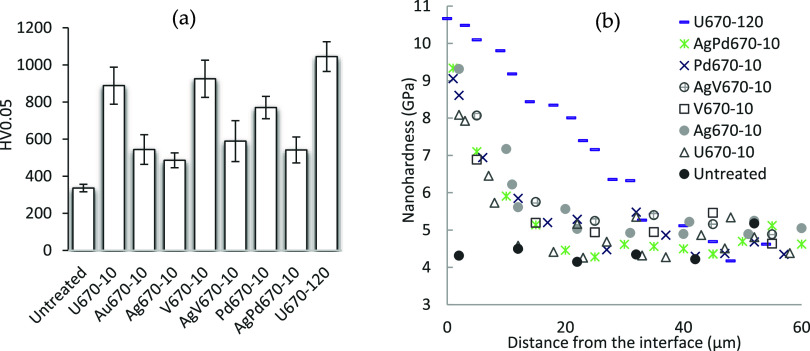
(a) Surface microhardness before and after CCT, and (b) Cross-sectional nanohardness profile under the oxide layer.

The untreated Ti6242 sample had a surface hardness of HV_0.05_336, and all of the surfaces were hardened after CCT as demonstrated in Figure 7a. After CCT at 670 °C, the surface hardness of Ti6242 increased significantly to HV_0.05_880 for U670–10 and HV_0.05_1045 for U670–120. The oxide layers with V (V670–10) and Pd (Pd670–10) demonstrated a hardness value similar to that of sample U670–10. The surface hardness of Au670–10 increased only to about HV_0.05_544, while those surfaces containing silver had even less hard surfaces with sample Ag670–10 hardened the least. As shown in the cross-sectional nanohardness measurements in Figure 7b, short-time treatment (10h) led to a shallow hardened zone (oxygen diffusion zone) for all the samples, while longer treatment time (120 h) generated a thick hardened zone up to 50 μm (U670–120).

### 3.5 Tribological Performance

**Figure 8:**
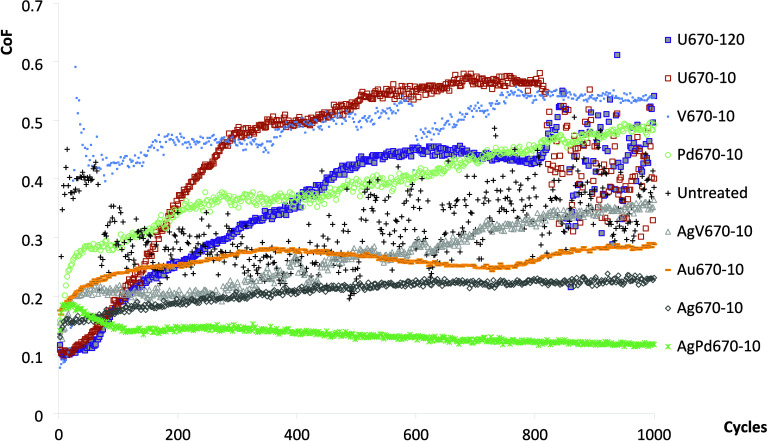
CoF change of the Ti6242 samples against an Ø8 mm WC ball.

The initial coefficient of friction (CoF) fluctuated around 0.4 in the first 100 cycles for the untreated Ti6242 sample, and it fell slightly to oscillate at about 0.3 for another 300 cycles before climbing up and becoming more unstable until the end of the test as seen in Figure 8.

**Figure 9:**
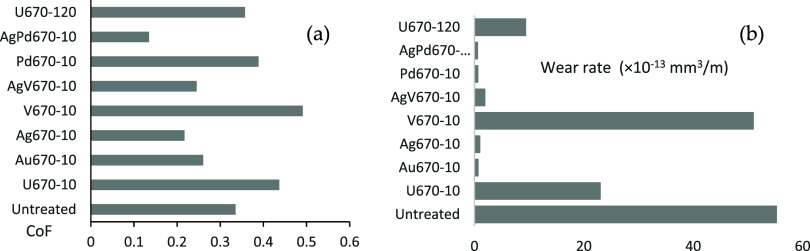
**T**ribological test comparisons: (a) average CoF and (b) wear rate.

The CoFs for samples U670–10 and U670–120 started low at about 0.1, and they both increased steadily and turned very unstable in the last 200 cycles. The CoF for sample U670–10 rose much faster and was higher than that of sample U670–120. Sample V670–10 had the highest initial CoF and remained high in the whole test range, although it dipped slightly in the first hundred cycles. The average CoF of V670–10 was the highest (0.49) among all the samples (Figure 9a). Sample Pd670–10 had a low CoF in the beginning, but it escalated steadily until the end of the test to about 0.49.

All the samples containing silver and gold had relatively low and steady CoF in the tribotest, although the initial CoFs were slightly higher at 0.1–0.2. For the sample AgV670–10, the CoF started at 0.16 and grew continually to about 0.36. The CoF of Au670–10 increased from about 0.20 to 0.28 at about cycle 300 and decreased slightly to 0.25 at cycle 700 before rising again gradually to 0.29 at the end of the test. The CoF of Ag670–10 rose gradually from 0.15 to about 0.23. For sample AgPd670–10, after an initial surge from 0.13 to 0.19 in the first 20 cycles, the CoF reduced slowly to 0.12 with the lowest average CoF of 0.14 (Figure 9a).

**Figure 10:**
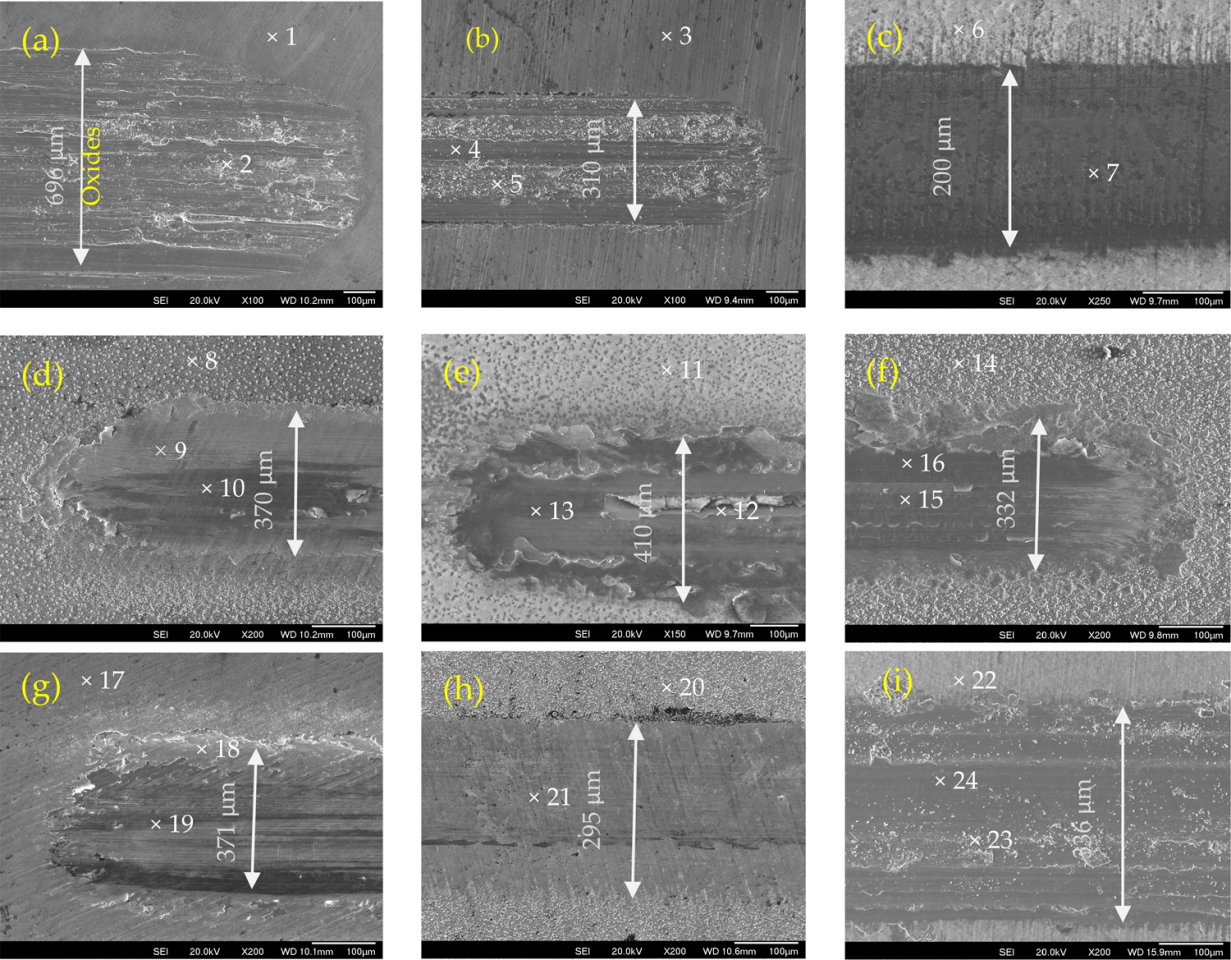
Wear tracks: (a) Untreated, (b) U670–10, (c) Au670–10, (d) Ag670–10, (e) V670–10, (f) AgV670–10, (g) Pd670–10, (h) AgPd670–10, and (i) U670–120.

After a 1000-cycle reciprocal test, a much wider (about 696 μm) and deeper track was created for the untreated sample (Figure 10a) with a wear area of 5518 μm^2^. Deep abraded grooves together with adhesive and oxidative areas were formed on the surface of the untreated sample, as identified by EDX (point 2 in Table 3). Although the width of the track on sample U670–10 was narrower than sample U670–120 (Figure 10b,i), the wear area was larger and the wear rate was higher (Figure 9b), possibly due to the oxide layer being cut through earlier. V670–10 had a track of 410 μm wide with a lot of delaminated layers in the track, resulting in a higher wear rate too. AgV670–10 had a much-reduced wear area with a narrower track (Figure 10f), while Ag and V were still identified in the track (points 15 and 16 in Table 3). The wear areas on the other samples (Ag670–10, Au670–10, AgPd670–10, and Pd670–10) were barely measurable, resulting in very low wear rates (Figure 9b). Au670–10 and AgPd670–10 had very smooth tracks (Figure 10c,h) and enough noble metals can still be picked up in the tracks by EDX.

**Table 3:** EDX Identification of Major Elemental Information at Different Locations of the Track

	at %	O	Al	Ti	Zr	Mo	Sn	W	Ag	Au	V	Pd
Untreated	1	1.9	6.61	88.86	1.36	0.77	0.5					
	2	3.87	8.32	86.02	1.16		0.48	0.11				
U670–10	3	5.89	5.98	85.85	1.19	0.61						
	4	14.21	6.18	77.11	1.24	0.72	0.48	0.06				
	5	25.54	5.09	67.97	0.89		0.38	0.13				
Au670–10	6	40.59	3.71	53.01	1.12	0.55	0.31			0.71		
	7	39.21	3.64	55.65	0.33		0.27	0.09		0.72		
Ag670–10	8	29.36	3.62	52.96	0.84	0.82			12.4			
	9	19.99	4.84	65.8	0.8			0.5	8.07			
	10	18.49	3.87	71.06	1.06	0.81	0.42	0.3	3.99			
V670–10	11	27.4	2.59	64.38	0.64		0.22				4.77	
	12	25.27	4.54	67.49	1.03	0.53	0.39				0.67	
	13	36.14	3.85	57.27	0.68	0.35		0.16			1.55	
AgV670–10	14	49.66	7.99	30.87	0.5	0.44	0.16		6.65		3.73	
	15	33.32	4.69	45.23	0.7	0.46		1.07	11.17		3.36	
	16	39.55	4.09	47.2	0.76	0.42		0.61	4.23		3.13	
Pd670–10	17	18.53	3.62	60.33	0.89	0.49						16.14
	18	27.47	3.88	65.22	1.19	1.05	0.44	0.13				0.62
	19	42.85	3.15	47.32	0.64		0.27	4.18				1.58
AgPd670–10	20	21.6	2.55	29.21	0.17	0.06			23.9			22.43
	21	0.87	3.48	58.24	0.23				27.53			9.64
U670–120	22	34.51	6.32	57.33	0.97	0.47	0.39					
	23	19.34	6.19	71.74	1.21	0.55	0.45	0.02				
	24	5.15	6.24	8588	1.4	0.55	0.47					

## 4 Discussion

Titanium has a high affinity to oxygen and a solid solubility of 14.5 wt %.^22^ Titanium alloys are easily oxidized even at room temperature, and this tendency increases with elevated temperatures and becomes more pronounced at temperatures above 600 °C. However, due to the addition of alloying elements, such as Mo, the oxidation of Ti6242 is slower than that of CPTi and Ti6Al4 V alloys.^20,23^ The thickness of the oxide layer formed on the Ti6242 surface was less than 1 μm after a long treatment of 120 h at 670 °C (Figures 4i and 5). A mixture of anatase and rutile phases appeared on the surface (Figure 6). Aging or annealing is commonly used to homogenize the structure and relieve internal stress in titanium alloys after working. Typically, the lower temperature and short treatment time are desirable to avoid the degradation of the mechanical strength.^24^

Gold particles promoted the outward diffusion of Ti atoms to react with oxygen and accelerate the ceramic conversion of Ti6242 into rutile oxides, similar to CPTi and Ti6Al4 V alloys.^13,25^ The mutual movement of oxygen and titanium atoms facilitated the uniform distribution of gold particles within the forming oxide film. In turn, this helped relieve internal stresses and refine the grain size, resulting in a more uniform, adherent, and compact oxide layer. Ag has a dramatic effect on boosting the oxidation of Ti6Al4 V but less effect on other titanium alloys like CPTi.^12^ The deposition of silver on the surface of Ti6242 tended to agglomerate on the surface rather than accelerate the oxidation (Figures 3d and 4d). Fewer oxides can be identified by XRD (Figure 6). Vanadium sped up the oxidation of Ti6242 remarkably, and the thickness of the oxide layer reached 2.5 μm, which was 10 times that of sample U670–10 and two times thicker than that of U670–120. Vanadium had a similar impact on the oxidation of CPTi as reported earlier.^15^ However, a lower concentration of oxygen in the oxide layer (Figure S3) indicated that vanadium facilitated oxygen diffusion to form a thicker oxygen-deficient oxide layer. Together with Ag, their compound effect becomes clearer. However, some regular and irregular blocks were formed on the surface (Figure 3e/f). Pd670–10 had a smooth surface and thick dual layers (Figures 3g and 4g). The superficial layer was composed of more Pd than the very dense and continuous underlayer, which might be due to the formation of PdO as identified by XRD (Figure 6). The higher content of Pd on the surface and in the top layer, as demonstrated in Figure S5, also provided evidence. AgPd670–10 also had dual layers with an irregular surface layer and a shallow underlayer (Figure 4h). Silver and palladium tended to agglomerate on the surface in the superficial layer (Figure S6), and some silver oxide can be identified, as shown in Figure 6. An oxygen diffusion zone can be seen under the oxide layer, which has less and smaller beta phase thanks to the dissolution and diffusion of the oxygen, an α-stabilizer (Figure 4). This corresponds to a shallow hardened zone under the oxide layer due to a short treatment time, as shown in Figure 7b.

As shown in Figure 5, regarding to the thickness of the oxide layer, V alone or together with silver has the most pronounced effect on the oxidation of Ti6242, and the surfaces are rougher, especially for the AgV670–10, due to the formation of the blocks. Ag/Pd or Pd has a remarkable impact but with dual layers produced on the surface of Ti6242. Au also has a notable effect with a dense oxide layer. The impact of silver is controversial, as it tends to agglomerate on the surface.

As shown in Figure 7a, the surface of Ti6242 samples becomes harder after CCT as ceramic titanium oxide layers have high hardness.^26^ U670/120 has the highest surface hardness, which is mainly composed of rutile, although the oxide layer is less than 1 μm, while a deeper oxygen diffusion zone with high nanohardness provides strong support (Figure 7b) as oxygen increases the hardness by solid solution strengthening.^27^ Sample V670–10 had a high hardness value due to the thicker oxide layer, while mixing with silver slightly reduced the surface hardness of AgV670–10. Similarly, the surface of Pd670–10 is harder than that of AgPd670–10. Ag670–10 has the weakest enforcement of the surface, and gold is better than silver. The samples treated for 10 h at 670 °C had a relatively shallow hardened diffusion zone in comparison to a 120-h treatment, as evidenced in Figure 7b.

A WC-Co cemented carbide ball has a hardness value equivalent to the TiO_2_ ceramic layer (HV 1100–1200), which is much harder than that of untreated Ti6242 (HV_0.05_336).^28^ The hard ball ploughed into the softer Ti6242 surface, leading to two-body abrasion, which created a wider and deeper track with some delaminated platelets and oxidative areas. The adhesive nature of the material resulted in material transfer and galling and thus a high and unsteady CoF (Figure 8). The cyclic loading led to subsurface crack initiation and propagation and thus resulted in material removal and delamination. A similar behavior was reported when Ti6242 slid against an AISI E52100 steel ball.^29,30^

The hard titanium oxide layer can reduce the friction and wear of titanium alloys due to the ceramic/ceramic contact (TiO_2_/WC), replacing the metal/ceramic contact (Ti/WC), which decreases the adhesive action.^31^ The maximum Hertzian contact pressure of the WC ball with untreated Ti6242 was about 1425 MPa, which increased to 2116 MPa for the ceramic-converted surface. Once the oxide layer was cut through, mixed abrasive and adhesive wear resulted in a sharp rise and unstable CoF, as shown in Figure 8 for U670–10 and U670–120. The hard delaminated oxide debris can cause three-body abrasion, which contributed to the high average CoFs of U670–10 and U670–120, although their wear areas were smaller.

Gold has been used as a solid lubricant^32^ and blended into a thin TiO_2_ layer to reduce friction and wear.^18,33^ The CoF of Au670–10 slightly increased and then remained stable throughout the test duration (Figure 8). Evenly dispersed gold nanoparticles in the surface region (Figure S1) may act as a barrier, preventing the formation of adhesive junctions. In the meantime, gold does not oxidize easily and may help stabilize the oxide layer, preventing excessive oxidation-driven wear and creating a more stable tribo-oxidation process with a low average CoF of 0.26. This effect was attributed to the ability of the gold particles to self-restore under the applied load due to their low shear strength.^34^ As gold particles were embedded in the oxide, they can enhance ductility and crack resistance and reduce material loss by preventing brittle fracture of the TiO_2_ layer. Further details on this phenomenon can be found in our earlier report.^21^

Silver has a low shear strength and acts as a solid lubricant,^35^ reducing the tendency of Ti to adhere to the WC ball, and lowering material transfer and wear rate. This can transform the wear mechanism from severe adhesion to milder oxidative wear, which notably reduced the CoF and wear area for Ag670–10, though the oxide layer was thin. The agglomeration of silver may lead to localized wear or create third-body abrasion (Figure 10d). A high average CoF and wear rate were observed on sample V670–10 (Figures 8 and 9), which might be due to the formation of hard and brittle vanadium oxides (such as V_2_O_3_ or VO_2_) or even alumina as the blocks on the surface were rich in Al, V, and O. This is also supported by the rough surface (Figure 2) and relative higher surface hardness (Figure 7a). The harder and rougher oxide layer could lead to more mechanical interlocking and higher plowing resistance, which increases friction. The brittle nature of the oxide film is easy to break apart and delaminated under reciprocating sliding, leading to third-body wear (Figure 9e).^36^ A relatively poor tribological performance was also found on the CCTed CPTi sample with vanadium.^15^ When Ag was added together with V, the CoF of sample AgV670–10 was reduced, and the wear rate decreased markedly compared to that of V670–10. This might be due to the lubricating nature of Ag, and the incorporation of Ag also reduced the hardness of the oxide layer, which made it less brittle, although it became rougher.

Palladium helped create dual layers on Pd670–10, as seen in Figure 4g, and the surface was smooth (Figures 2 and 3g). EDX identification on the top layer indicated a high Pd content, which modifies the titanium oxide structure, as verified by the XRD examination in Figure 6. The steady increase in CoF might be due to the counterpart ball gradually cutting through and breaking the superficial layer with medium hardness, which leads to mild third-body wear. Pd was still detectable in the track (Locations 18/19 in Figure 10g) after the 1000 cycle test, indicating the oxide layer was not fully removed. The gradually increased CoF suggested that Pd does not act as a solid lubricant. Adding silver together with Pd, the lowest friction and wear rate were recorded for sample AgPd670–10, thanks to the lubrication effect of silver and palladium, as both high amounts of Ag and Pd can still be seen in the track as indicated in spot 20/21 in Figure 10h.

## 5 Conclusions

In this work, we predeposited various metal layers (Au, Ag, V, Ag/V, Pd, Ag/Pd) onto the surface of Ti6242 before the CCT to compare their impact on the treatment to obtain an efficient process. The following conclusions can be drawn:

- CCT of Ti6242 at 670 °C for 120 h resulted in the formation of a compact oxide layer of less than 1 μm thickness, providing the surface with high hardness and good tribological properties until the oxide layer was worn away.
- Vanadium proved to be the most effective in boosting the oxidation of Ti6242. However, the resulting oxide layer exhibited poor quality, leading to inferior tribological performance. Palladium accelerated the treatment slightly, but it led to the formation of dual layers, increasing friction and reducing wear resistance. While silver had limited effectiveness in promoting oxidation, it did contribute to reducing friction and wear. Furthermore, the addition of Ag into V or Pd decreased the CoF of CCTed Ti6242 samples and improved their wear resistance, although the quality of the oxide layer needed enhancement.
- Gold expedited the CCT of Ti6242 alloy, resulting in a compact oxide layer containing evenly dispersed gold particles in the top layer, which enhanced the friction condition and wear resistance of the material.

With the assistance of the predeposited metal layer, a 10 h treatment could generate an oxide layer with better tribological performance compared to the traditional 120 h treatment (U670–120), significantly shortening the treatment time of CCT. However, further work is required to optimize the amount of the metallic layer, the treatment temperature, and the duration to enhance the quality of the oxide and their performance.

## Data Availability

Al the data available in this paper and the supplement document.

## Supporting Information Available

Detailed EDX analysis of the surface and cross-section of samples Au670–10, Ag670–10, V670–10, AgV670–10, Pd670–10, AgPd670–10, and U670–120; and an evolution of surface phase constituent on Ti6242 without predeposited metal layer also presented (PDF)
