# Supplementary material for: Impact of Catalytic Metals on the Ceramic Conversion Treatment of Ti-6Al-2Sn-4Zr-2Mo Alloy
Source: ACS Omega. 2025 Mar 10;10(11):11534–43. doi: 10.1021/acsomega.5c00209 (PMC11947812; doi:10.1021/acsomega.5c00209)
Supplement: Supplementary file 1 — ao5c00209_si_001.pdf [file ao5c00209_si_001.pdf]

Impact of catalytic metals on the ceramic conversion treatment of Ti-6Al-2Sn-4Zr-2Mo alloy

Zhenxue Zhang [1*], Xiaoying Li [1] and Hanshan Dong [1]

[1] School of Metallurgy and Materials, The University of Birmingham, UK B15 2TT;
* Correspondence: z.zhang.1@bham.ac.uk

Supplement Figures

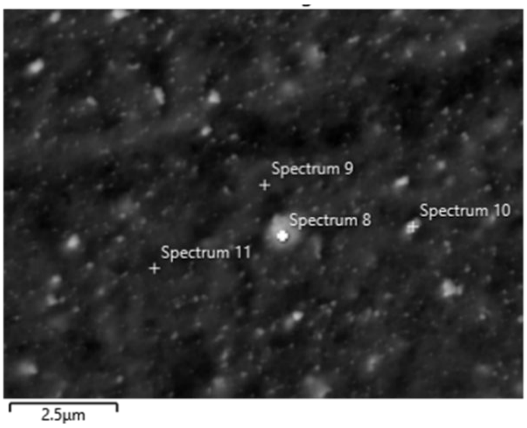

At %	8	9	10	11
**O**	45.13	28.61	45.06	26.79
**Al**	4.53	4.49	4.12	4.78
**Ti**	41.82	66	48.7	66.32
**Zr**	0.71	0.46		1.16
**Sn**	0.34	0.44	0.3	0.38
**Au**	7.47		1.82	0.57

Figure S1. Surface of sample Au670-10 and its EDX composition

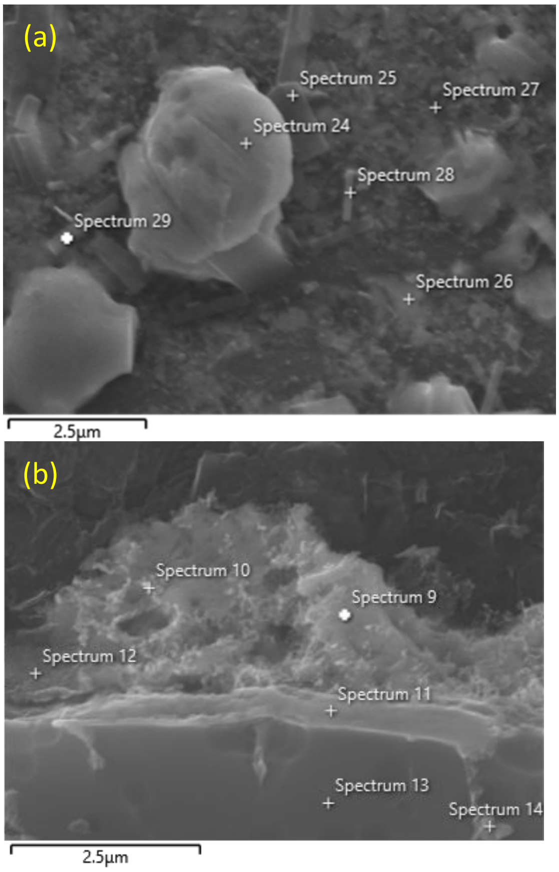

At%	24	25	26	27	28	29
**O**	3.44	25.65	19.8	20.38	22.05	29.36
**Al**	1.04	3.94	3.73	4.2	4.66	2.76
**Ti**	6.29	64.89	46.2	68.9	69.32	52.96
**V**			0.65			0.86
**Zr**		1.07	0.76	1.09	1.12	0.84
**Mo**		0.55	0.56	0.64	0.47	0.82
**Ag**	89.23	3.49	28.3	4.39	1.95	12.3
**Sn**		0.41	100	0.39	0.43	
**Ta**						0.1

At%	9	10	11	12	13	14
**O**	11.28	13.79	38.38	39.38	5.7	8.97
**Al**	1.94	2.68	2.94	2.35	6.43	6.62
**Ti**	8.01	10.95	51.13	46.11	85.39	81.41
**Zr**	0.12	0.13	0.89	0.95	1.22	1.31
**Mo**	0.25	0.58	0.43	0.45	0.51	1.24
**Ag**	78.4	71.63	5.72	10.06	0.3	
**Sn**	0	0.24	0.37	0.33	0.45	0.46
**Ta**			0.14	0.38		

Figure S2. Sample Ag670-10 and its EDX composition (a) Surface and (b) Cross-section

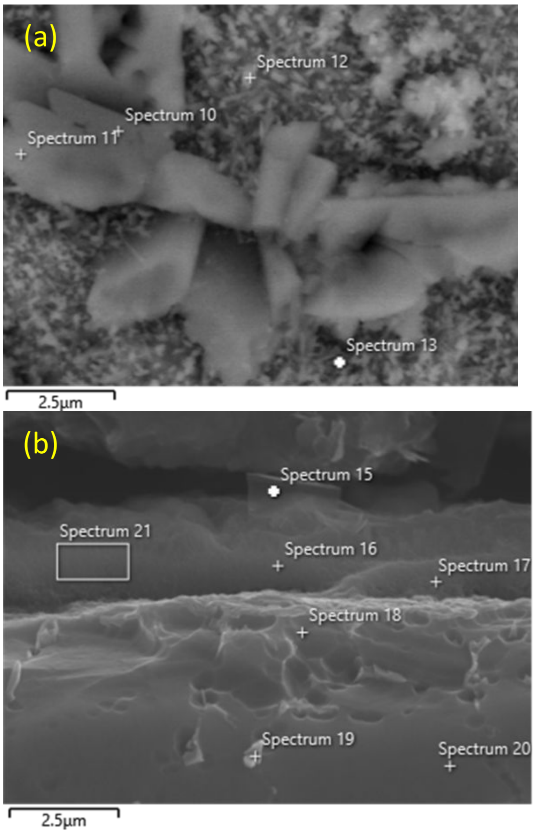

At %	10	11	12	13
**O**	19.25	32.59	27.4	20.83
**Al**	16.74	19.64	2.59	3.5
**Ti**	39.92	22.58	64.4	69.61
**V**	23.15	24.48	4.45	4.27
**Zr**	0.42	0.38	0.64	1
**Sn**	0.23	0.15	0.22	0.35
**Ta**	0.29	0.18	0.32	0.43

At %	15	16	17	18	19	20	21
**O**	18.79	33.52	6.03	4.6	6.81	5.55	27.05
**Al**	2.38	2.03	0.39	2.63	5.47	7.05	2.27
**Ti**	47.29	59.63	88.76	89.58	83.12	85.42	64.16
**V**	30.42	3.82	4.27	0.44	0	0.13	5.61
**Zr**	0.08	0.45	0.07	0.98	1.37	1.18	0.36
**Mo**	0.46	0.12	0.08	1.18	2.67	0.2	0.2
**Sn**	0.58	0.29	0.41	0.58	0.56	0.46	0.35
**Ta**	100	0.12	100		100	100	100

Figure S3. Sample V670-10 and its EDX composition (a) Surface and (b) Cross-section

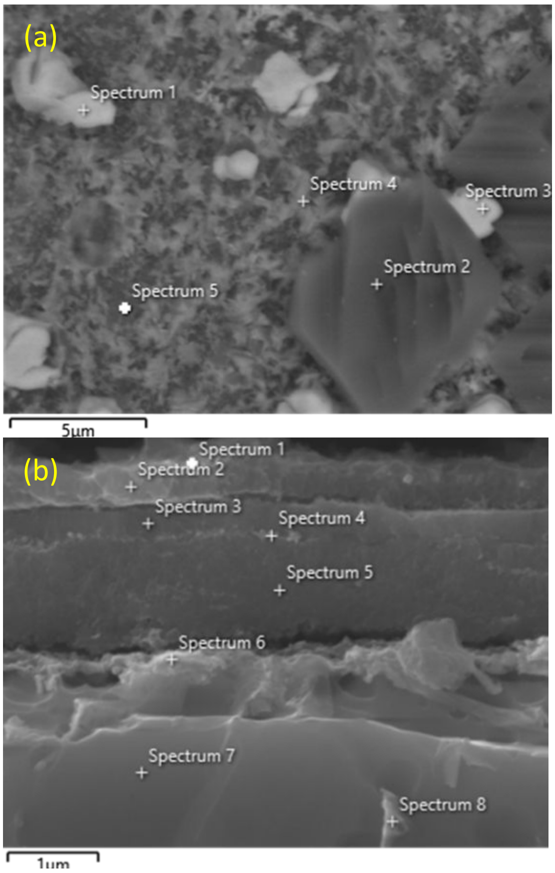

At %	1	2	3	4	5
**O**	11.8	40.15	10.99	31.79	34.54
**Al**	3.09	0.77	1.25	10.88	7.09
**Ti**	7.01	54.67	14.33	34.29	47.7
**V**	0.91	1.18	0.58	8.31	3.93
**Zr**	0.08	0.37	0.24	0.39	0.64
**Mo**				0.64	0.73
**Ag**	77.1	2.86	72.61	13.7	5.2
**Ta**					0.09
**W**					0.08

At%	1	2	3	4	5	6	7	8
**O**	45.18	48.32	32.91	37.3	36.92	37.41	9.56	10.93
**Al**	9	5.05	4.33	4.88	3.04	5.2	6.41	6.93
**Ti**	32.99	40.75	56.11	50.77	55.96	49.77	81.45	76.7
**V**	0.84	0.72	1.02	0.78	0.77	0.37	0	0.19
**Zr**	0.53	0.58	0.89	0.85	0.98	1.08	1.2	1.36
**Mo**	0.43	0.32	0.3	0.31	0.36	1.44	0.74	3.29
**Ag**	10.87	4.04	3.82	4.78	1.76	4.25	0.15	0.05
**Sn**	0.17	0.22	0.41	0.33	0.22	0.48	0.49	0.55
**Ta**			0.11					
**W**			0.1					

Figure S4. Sample AgV670-10 and its EDX composition (a) Surface and (b) Cross-section

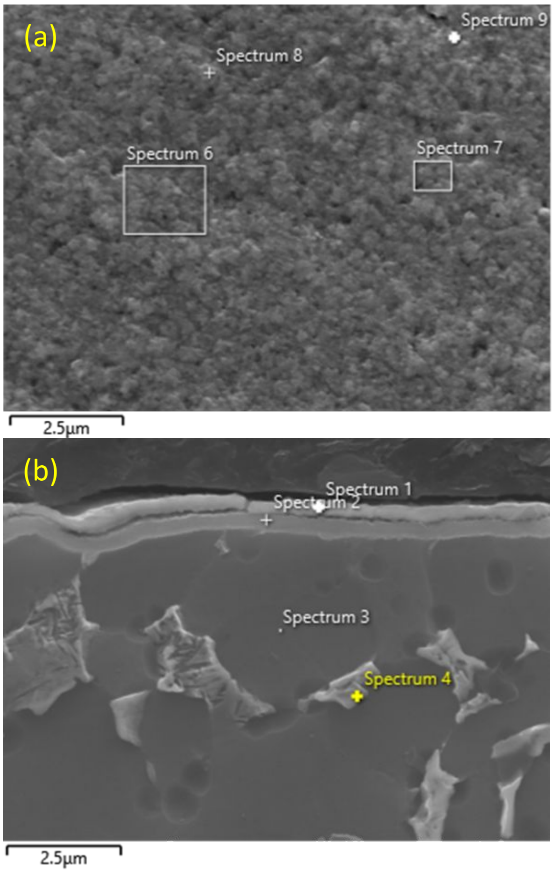

At %	6	7	8	9
O	15.43	16.36	18.53	17.89
Al	3.67	4.22	3.62	3.45
Ti	65.69	64.52	60.33	61.28
Zr	0.99	0.85	0.89	1.12
Mo	0.7	0.25	0.49	0.98
Pd	13.52	13.8	16.14	15.16
Ta				0.11

At %	1	2	3	4
O	10.77	24.36	4.53	6.49
Al	4.96	4.57	5.91	6.62
Ti	45.13	61.41	86.83	82.1
Zr	0.72	1.04	1.34	1.26
Mo	0.8	0.59	0.78	2.9
Pd	36.18	7.56		
Sn	1.15	0.47	0.49	0.64
Ta	0.3		0.12	

Figure S5. Sample Pd670-10 and its EDX composition (a) Surface and (b) Cross-section

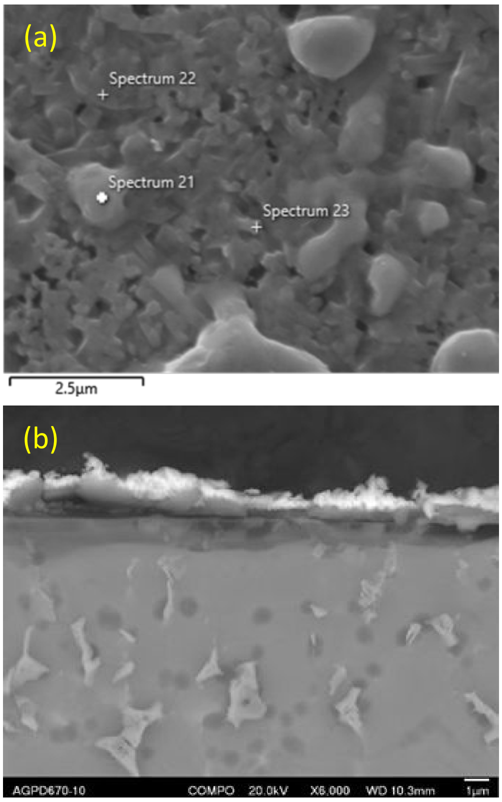

Wt%	21	22	23
O	5.36	21.6	20.13
Al	2.09	2.55	3.15
Ti	4.03	29.21	43.28
Zr	0.18	0.17	0.48
Mo	0.47	0.06	0.32
Pd	13.17	22.43	22.05
Ag	74.69	23.9	10.59
Pt		0.08	

Figure S6. Sample AgPd670-10 (a) Surface SEM and its EDX composition and (b) Backscattering image of the cross-section

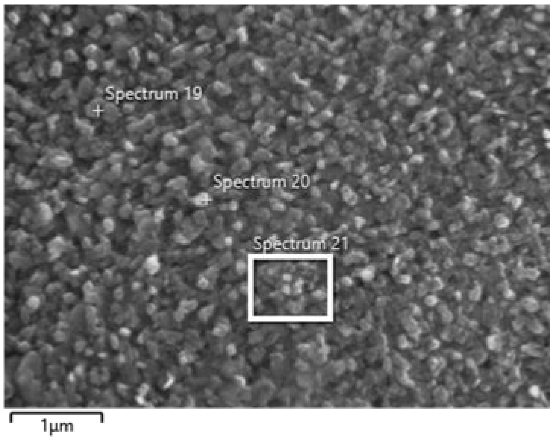

At%	19	20	21
O	55.57	54.02	62.96
Al	3.83	4.04	3.48
Ti	39.77	41	32.07
Zr	0.62	0.72	0.74
Mo			0.59
Sn	0.22	0.23	0.17

Figure S7. Surface of sample U670-120 and its EDX composition

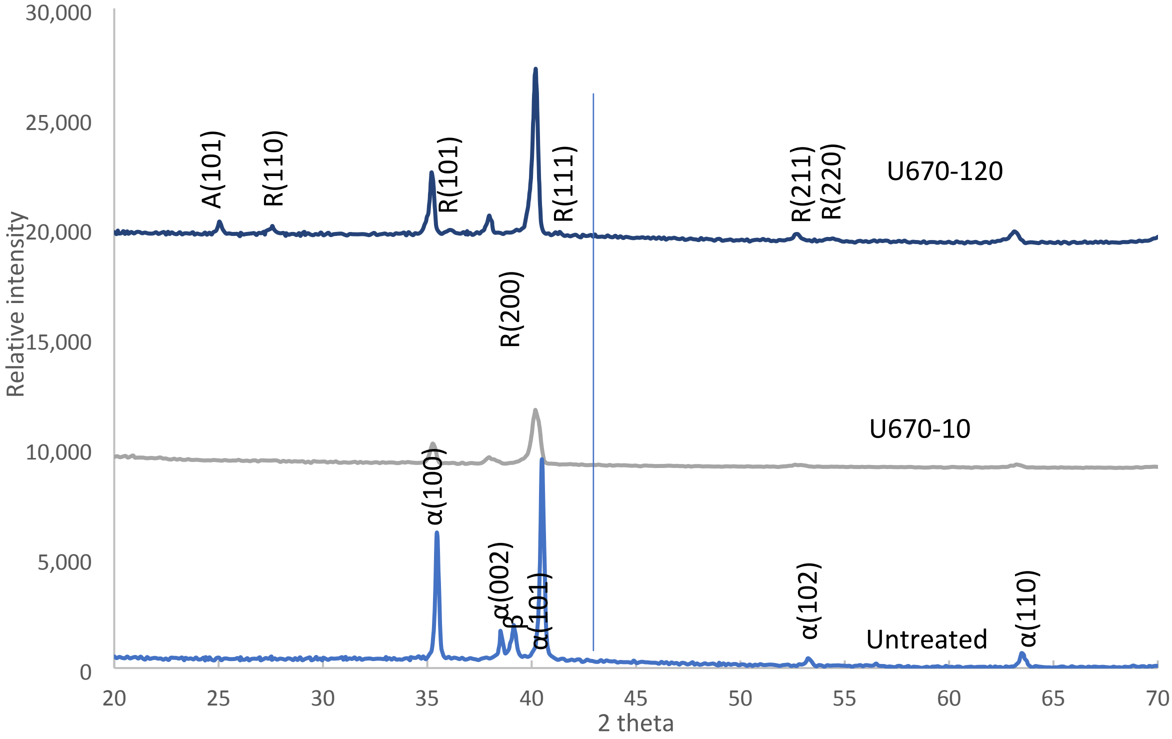

Figure S8. Evolution of surface phase constituents on Ti6242 without any addition metal layer CCTed at 670°C for 10 and 120 hours.
